# Mapping the role of macro and micronutrients in bone mineral density: a comprehensive Mendelian randomization study

**DOI:** 10.1007/s00394-025-03665-2

**Published:** 2025-04-16

**Authors:** Rong Xiang, Xunying Zhao, Linna Sha, Mingshuang Tang, Xueyao Wu, Li Zhang, Jiaojiao Hou, Qin Deng, Yang Qu, Jiangbo Zhu, Chenjiarui Qin, Changfeng Xiao, Jinyu Xiao, Yangdan Zhong, Bin Yang, Xin Song, Jinyu Zhou, Tao Han, Sirui Zheng, Ting Yu, Jiaqiang Liao, Mengyu Fan, Jiayuan Li, Zhonghua Liu, Xia Jiang

**Affiliations:** 1 Department of Nutrition and Food Hygiene, West China School of Public Health and West China Fourth Hospital, Sichuan University, Chengdu, Sichuan China; 2 Department of Epidemiology and Health Statistics, West China School of Public Health and West China Fourth Hospital, Sichuan University, Chengdu, Sichuan China; 3 Department of Clinical Nutrition, No.4 West China Teaching Hospital, Sichuan University, Chengdu, Sichuan People’s Republic of China; 4 Department of Clinical Neuroscience, Center for Molecular Medicine, Karolinska Institutet, Solna, Stockholm Sweden

**Keywords:** Nutrients, Bone mineral density, Mendelian randomization, Antioxidants, Vitamin B12, Selenium

## Abstract

**Background:**

Macro and micronutrients may play an important role in osteoporosis development; however, observational studies have yielded inconsistent results. Clarifying these associations is vital to the development of nutritional recommendations aimed at preventing osteoporosis.

**Methods:**

Utilizing the largest available genome-wide association study (GWAS) summary statistics to date, we performed a two-sample Mendelian randomization (MR) analysis to investigate the causal effects of energy-adjusted macronutrient intake (fat, protein, carbohydrate, and sugar) and circulating levels of 20 micronutrients (ten each for vitamins and minerals) on heel estimated bone mineral density (eBMD), a promising marker for osteoporosis risk and fracture susceptibility. Sensitivity, sex-specific, and one-sample MR analyses were applied to further validate and annotate the results.

**Results:**

Among all nutrients, four genetically predicted circulating levels of micronutrients were suggestively associated with eBMD (vitamin A: $\beta$_IVW_ = − 0.054, *P*_IVW_ = 3.70 × 10^–2^; vitamin B12: $\beta$_IVW_ = − 0.020, *P*_IVW_ = 3.71 × 10^–6^; vitamin E: $\beta$_IVW_ = 0.277, *P*_IVW_ = 3.22 × 10^–2^; selenium: $\beta$_IVW_ = 0.023, *P*_IVW_ = 5.37 × 10^–3^; all *P*_intercept_ > 0.05). All these results were also considered robust, as sensitivity analyses yielded directionally consistent results. However, only the causal effects of vitamin B12 and selenium on eBMD remained significant after Bonferroni correction and were not confounded by obesity, smoking status, or alcohol consumption. Sex-specific analysis revealed a male-specific causal association between vitamin E and eBMD ($\beta$_IVW_ = 0.275, *P*_IVW_ = 9.81 × 10^–14^). Additionally, using individuallevel data from the UK Biobank cohort, one-sample MR analysis found no causal relationships between diet-derived nutrient intake and eBMD in the overall population, as well as in the females or the males.

**Conclusions:**

Our findings suggest that appropriate levels of plasma vitamin B12 and adequate levels of serum selenium are crucial for delaying bone loss and promoting bone health, emphasizing the need for nutritional recommendations to maintain optimal levels of these nutrients to promote eBMD and prevent osteoporosis.

**Supplementary Information:**

The online version contains supplementary material available at 10.1007/s00394-025-03665-2.

## Introduction

Osteoporosis, characterized by a decrease in bone mineral density (BMD), is a common chronic disease that causes substantial health and socioeconomic burdens worldwide [1, 2]. Bone metabolism is highly responsive to nutrients, including macro and micronutrients, all of which affect bone growth and bone loss [3]. While macronutrients are pivotal for energy and tissue structure, their exact role in BMD remains inconclusive. For instance, an umbrella review indicated “insufficient” evidence to support a link between high protein intake and BMD at various sites, including the hip and the spine [4]. Similarly, micronutrients, which are crucial for health despite their minimal caloric contribution, also show inconsistent effects on BMD according to observational studies. For example, one cross-sectional study with 1465 participants reported 6.2% higher lumbar spine BMDs in participants with vitamin B12 concentrations ≥ 148 pmol/L, even after adjusting for protein intake and plasma homocysteine [5], whereas another study with 2806 participants found no significant association [6]. These inconsistencies might stem from variations in study design or population characteristics, as well as limitations inherent in observational studies including the potential impact of confounders or reverse causality.

Mendelian randomization (MR) is a method that utilizes single nucleotide polymorphisms (SNPs) fixed at conception as instrumental variables (IVs) to reveal causality [7]. This method effectively minimizes the influence of confounding factors and reverse causation. Currently, several MR studies have been conducted to elucidate the causal relationships between nutrients and bone-related traits [8–13], nevertheless, these studies have only examined a small number of nutrients while neglecting important nutrients that are also critical for bone health, such as macronutrients, vitamin K1, and manganese. Additionally, regarding bone-related traits, ultrasound-derived BMD at the heel calcaneus (referred to as heel estimated BMD, eBMD) holds an irreplaceable position [14]. Specifically, eBMD demonstrates the highest heritability (50% ~ 80%) [15–19], has the largest available genome-wide association study (GWAS) summary statistics [20], exhibits an 84% overlap in genome-wide significant loci with dual-energy X-ray absorptiometry (DXA)-derived BMDs, and shows moderate correlations in effect sizes with DXA-derived BMDs (Pearson’s *r* = 0.69 for lumbar spine and 0.64 for femoral neck) [14]. All of these characteristics allow for a better prediction of osteoporosis diagnosis and fracture risk. However, no MR study has comprehensively investigated the causal relationships between a broad spectrum of nutrients and eBMD using the hitherto largest available GWAS summary statistics and IVs. The only existing four MR studies on this topic covered a small set of nutrients, with 1, 3, or 6 nutrients per each study, encompassing a total of five vitamins and five minerals. Moreover, the number of IVs in these studies ranged from 1 to 14, accounting for 0.8% to 6.0% of the phenotypic variation [8, 9, 12, 13]. Besides, two of these studies utilized GWAS summary statistics for eBMD involving 142,487 [13] and 394,929 participants [12], respectively. With the advent of larger GWAS summary statistics and an increased number of IVs, there is an opportunity to explore causal associations between nutrients and eBMD with greater power. For instance, serum calcium now has 208 IVs accounting for 5.7% of the variation [21] (vs. the previous 7 IVs accounting for 0.8% of the variation [22]); the GWAS summary statistic for eBMD now includes 426,824 participants [20] (vs. the previous 142,487 [14] or 394,929 [23] participants). In addition, several key confounders—obesity, smoking status, and alcohol consumption—can now be accounted for in the nutrients-eBMD relationship through the extension of conventional MR approaches (multivariable MR [24]), a consideration that previous studies did not address.

**Fig. 1:**
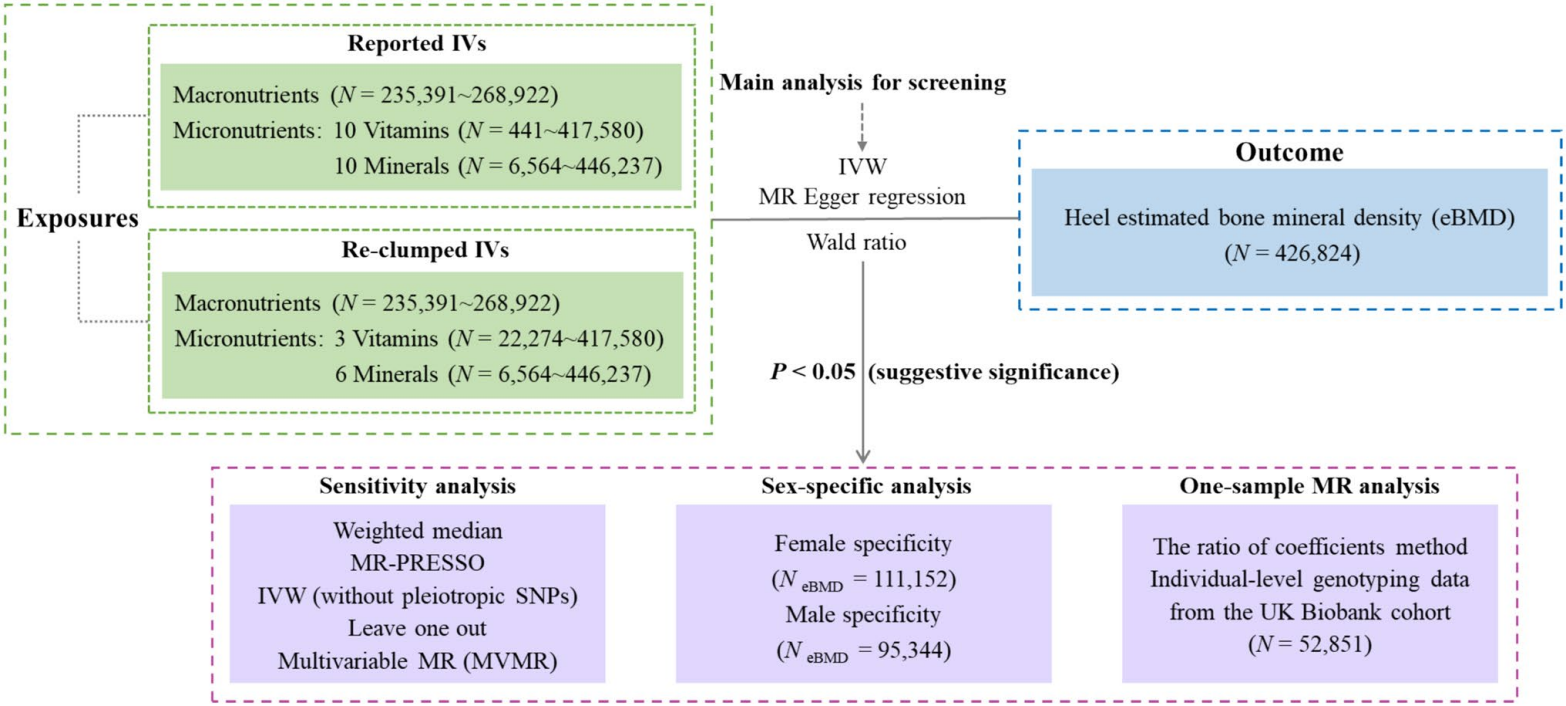
Overall study design of the comprehensive Mendelian randomization analysis. *IVs* instrumental variables, *N* sample size, *IVW* inverse variance weighted, *MR* Mendelian randomization, *MR-PRESSO* MR-Pleiotropy Residual Sum and Outlier, *SNPs* single nucleotide polymorphisms. Clump parameter of reported IVs: *P*-threshold < 2.2 × 10^–9^ ~ 1 × 10^–6^; *r*^*2*^ < 0.01 ~ 0.3 within a 0.20 ~ 10 Mb window. Clump parameter of reported IVs: *P*-threshold < 5 × 10^–8^; *r*^*2*^ ≤ 0.001 within a 10 Mb window

Therefore, leveraging the largest GWAS summary statistics to date, we aimed to: (I) systematically and comprehensively explore the causal effects of energy-adjusted macronutrient intake (fat, protein, carbohydrate, and sugar) and circulating levels of 20 micronutrients (ten each for vitamins and minerals) on eBMD; (II) examine their independent causal effects after accounting for the confounding effects from potential confounders; (III) investigate their sex-specific causal effects on eBMD; and (IV) supplement information using diet-derived nutrients collected via online 24-h dietary recall (24HDR) in the UK Biobank cohort. The overall study design is shown in Fig. 1.

## Materials and methods

This study adhered to the guidelines of the Strengthening the Reporting of Observational Studies in Epidemiology—Mendelian Randomization (STROBE-MR) [25] (Supplementary File 1, https://www.strobe-mr.org/). All GWASs included were approved by the relevant ethics committees and all participants provided written informed consent in their original studies. More details can be found in each original GWAS.

### Summary-level data for macro and micronutrients

We systematically searched the GWAS Catalog and the PubMed, selecting GWAS with the largest sample size in the European population for each exposure. Briefly, GWAS for macronutrients was conducted by Meddens and colleagues [26], which included individuals aged 27 to 71 years and excluded those on calorie- or macronutrient-restricted diets. All cohorts in the GWAS used self-report questionnaires containing ≥ 70 food items and reported similar average intakes. The analysis of energy-adjusted fat, protein, and carbohydrate intake included 268,922 participants, while the analysis of energy-adjusted sugar intake included 235,391 participants. GWASs for micronutrients primarily focused on objectively measured circulating levels of ten vitamins and ten minerals. GWASs for lycopene [27], beta-carotene [28], vitamin A (retinol) [29], vitamin B6 [30], vitamin B9 (folate) [31], vitamin B12 [31], vitamin C (ascorbate) [32], vitamin D [33], vitamin E (alpha-tocopherol) [34], and vitamin K1 (phylloquinone) [35] included 441 ~ 417,580 individuals, while GWASs for calcium [21], phosphorus [36], sodium [37], potassium [37], magnesium [38], selenium [39], iron [40], copper [41], zinc [42], and manganese [42] included 6,564 ~ 446,237 individuals. More details of included GWASs for each nutrient can be found in Table 1.

**Table 1:** Details of reported genetic instruments for investigated nutrients

Exposure	PMID	Year	Sample size	Number of IVs	*R*^*2*^	Mean *F*-statistic	Measurement	Clump parameters^#^	Summary statistics’ availability^#^
Macronutrients: energy-adjusted intake									
Fat	32,393,786	2021	268,922	6	0.002	66.40	FFQ/24HDR	r^2^ < 0.01, 0.5 MB, *P* < 5 × 10^–8^	Available
Protein				7	0.002	53.03			
Carbohydrate				13	0.002	39.34			
Sugar			235,391	10	0.002	46.57			
Micronutrients: vitamins									
Lycopene	26,861,389	2016	441	5	0.310	29.09	Serum	*P* < 1 × 10^–6^	NA
Beta-carotene	19,185,284	2009	3,881	1	0.025	98.62	Plasma	r^2^ < 0.2, *P* < 5 × 10^–7^	NA
Vitamin A (retinol)	38,374,065	2024	17,268	8	0.022	47.28	Plasma	r^2^ < 0.1, 0.25 MB, *P* < 5 × 10^–8^	Available
Vitamin B6	19,303,062	2009	1,864	1	0.014	26.79	Plasma/Serum	*P* < 5 × 10^–8^	NA
Vitamin B9 (folate)	23,754,956	2013	37,341	2	0.008	141.91	Serum	*P* < 2.2 × 10^–9^	NA
Vitamin B12			45,576	11	0.051	213.63			
Vitamin C (ascorbate)	33,203,707	2021	52,018	11	0.018	84.81	Plasma	*P* < 5 × 10^–8^	Available
Vitamin D	32,242,144	2020	417,580	143	0.059	173.96	Serum	r^2^ < 0.01, 10 MB, *P* < 5 × 10^–8^	Available
Vitamin E (alpha-tocopherol)	21,729,881	2011	7,781	3	0.004	11.33	Serum	*P* < 5 × 10^–8^	NA
Vitamin K1 (phylloquinone)	25,411,281	2014	2,138	5	0.065	27.99	Plasma	r^2^ < 0.2, 0.20 MB, *P* < 5 × 10^–7^	NA
Micronutrients: minerals									
Calcium	33,462,484	2021	325,659	208	0.057	89.43	Serum	*P* < 5 × 10^–9^	Available
Phosphorus	20,558,539	2010	16,264	7	0.016	36.50	Serum	*P* < 4 × 10^–7^	NA
Sodium	31,409,800	2019	446,237	50	0.005	47.84	Urine	r^2^ < 0.1, 1 MB, *P* < 1 × 10^–8^	Available
Potassium			446,230	13	0.001	44.06			
Magnesium	20,700,443	2010	23,829	6	0.015	57.83	Serum	*P* < 5 × 10^–8^	NA
Selenium	25,343,990	2015	9,639	12	0.085	67.98	Toenail/Plasma/Whole blood	r^2^ < 0.3, *P* < 5 × 10^–8^	NA
Iron	33,536,631	2021	163,511	16	0.028	285.37	Serum	r^2^ < 0.1, 0.5 MB, *P* < 3 × 10^–8^	Available
Copper	34,523,676	2022	6,937	2	0.016	54.88	Serum/Whole blood/Erythrocyte	*P* < 5 × 10^–8^	NA
Zinc	38,594,418	2024	6,564	3	0.045	99.92	Whole blood	r^2^ < 0.2, *P* < 5 × 10^–8^	Available
Manganese				10	0.161	110.37			

*IVs* instrumental variables, *GWAS* genome-wide association study, *FFQ* food-frequency questionnaire, *24HDR* 24-h dietary recall, *NA* not available

^#^In their original GWASs

### Summary-level data for eBMD

The hitherto largest GWAS of eBMD was conducted by Morris and colleagues on 426,824 European participants aged 39 ~ 74 years from the UK Biobank [20]. eBMD was evaluated by quantitative ultrasound speed of sound (SOS) and broadband ultrasound attenuation (BUA) using a Sahara Clinical Bone Sonometer (Hologic Corporation, Bedford, MA, USA). The specific formula used to derive eBMD is: $eBMD=0.002592\times (BUA+SOS)-3.687$. eBMD values were expressed as mean $\pm$ standard deviation (SD), with an average of 0.51 $\pm$ 0.11 g/cm^2^ for females and 0.56 $\pm$ 0.12 g/cm^2^ for males. The genetic estimates were adjusted for age, sex, genotyping array, assessment center, and ancestry informative principal components 1 to 20. Considering that the physiological effects of nutrients may be influenced by sex, separate GWAS summary statistics for sex-specific eBMD were obtained from the UK Biobank dataset (http://www.nealelab.is/uk-biobank), which included 111,152 females and 95,344 males of European ancestry, respectively.

### Summary-level data for confounders

Three key factors, including obesity, smoking status, and alcohol consumption, were considered potential confounders in the nutrients-eBMD relationship. The hitherto largest GWAS for body mass index (BMI), a common indicator of overall obesity, was conducted by a collaboration of the UK Biobank (UKB) and the Genetic Investigation of Anthropometric Traits (GIANT) consortium, involving 806,834 adults of European ancestry [43]. For smoking status and alcohol consumption, we obtained the hitherto largest GWAS summary data from the GWAS & Sequencing Consortium of Alcohol and Nicotine, comprising 632,802 individuals for smoking initiation and 537,349 individuals for alcohol consumption, all of European ancestry [44].

### Selection of genetic instruments

For all macro and micronutrients, we collected previously reported independent index SNPs according to their original GWASs (*P*-threshold < 2.2 × 10^–9^ ~ 1 × 10^–6^; *r*^*2*^ < 0.01 ~ 0.3 within a 0.20 ~ 10 Mb window). Details of included IVs for each nutrient can be found in Table 1 and Supplementary Table 1. These IVs are termed “reported” IVs.

In addition, we clumped and identified independent IVs that reached genome-wide significance (*P*-threshold < 5 × 10^–8^) following a stricter and standard criterion (*r*^*2*^ ≤ 0.001 within a 10 Mb window). These IVs were termed “re-clumped” IVs and were used for additional analysis (Supplementary Table 3** ~ **4). Such a process was conducted for all macronutrients (energy-adjusted intake of fat, protein, carbohydrate, and sugar) and nine micronutrients (including three vitamins, i.e., vitamin A, vitamin C, vitamin D, and six minerals, i.e., calcium, sodium, potassium, iron, zinc, manganese), for which full-set GWAS summary statistics were made publicly available.

For both sets of IVs, the *F*-statistic was calculated to evaluate instrument strength, and an *F*-statistic of less than 10 indicated a weak instrument [45]. Statistical power was calculated with a web-based application (https://sb452.shinyapps.io/power/) [46]. The heterogeneity across the genetic instruments was evaluated by Cochran’s Q-statistics [47].

### UK Biobank cohort data

The UK Biobank cohort is a prospective, population-based study with deep genetic and phenotypic data collected from 502,364 individuals aged 40 ~ 69 years, all of whom provided electronically signed consent at recruitment. Genotyping was conducted by the Affymetrix Research Services Laboratory, resulting in genotype calls for 489,212 samples at 812,428 unique markers. More details have been published in previous studies [48]. At baseline, nutrient intake information was collected from 70,655 individuals using an online 24-h dietary recall (24HDR). The UK Biobank data used in this study was obtained under Application #99713. Finally, we included 52,851 individuals of white European ancestry with complete baseline data on diet-derived nutrient intake and eBMD.

## Statistical analysis

### Main analysis for screening

To detect putative causal effects of macro and micronutrients on eBMD, we initially performed a preliminary two-sample MR analysis to identify the key nutrients. These nutrients were to be subjected to further analyses. Nutrients that reached a *P* < 0.05 (suggestive significance) in any of the following three “screening” strategies were included in further analyses: (I) the inverse-variance weighted (IVW) approach (*P*_IVW_ < 0.05 [suggestive significance]) supplemented by the MR-Egger regression (*P*_intercept_ < 0.05 [no directional pleiotropy]), were applied to all nutrients that were with multiple reported IVs (Supplementary Table 1). The IVW approach [49] provides reliable estimates by assuming all IVs are valid, while the MR-Egger regression [50] accounts for directional pleiotropy by incorporating an intercept term; (II) the Wald ratio method was applied to nutrients with a single reported IV, i.e., vitamin B6 and beta-carotene (Supplementary Table 1); (III) the same strategy as in (I) was employed again for all macronutrients and nine micronutrients (including three vitamins and six minerals) with available re-clumped IVs (Supplementary Table 3).

### Sensitivity, sex-specific, and one-sample MR analyses

For nutrients with suggestive evidence of a causal effect on eBMD in the main analysis, we further performed an array of analyses including the sensitivity analysis, the sex-specific analysis, and the one-sample MR analysis to confirm and interpret the preliminary results.

We first performed several important sensitivity analyses to assess the robustness of results and to validate the MR model assumptions: (I) the weighted median approach [51] exhibits stronger robustness against invalid IVs, provided that less than half of the IVs are invalid; (II) the MR-Pleiotropy Residual Sum and Outlier (MR-PRESSO) approach [52] identifies and removes outlying IVs to provide unbiased estimates, assuming the remaining IVs are valid; (III) the IVW approach (without pleiotropic SNPs) removes pleiotropic IVs associated with potential confounders to control for pleiotropy according to the GWAS catalog (https://www.ebi.ac.uk/gwas/); (IV) the leave-one-out analysis identifies outlying IVs to ensure robust results by removing one IV at a time and performing IVW on the remaining IVs. A causal estimate was considered robustly significant if it was at least suggestively significant in any of the above three “screening” strategies and remained directionally consistent across all approaches used for sensitivity analyses.

Considering that BMI, smoking status, and alcohol consumption are recognized as major confounders in the nutrients-eBMD relationship, we applied a multivariable MR (MVMR) analysis [24] to estimate the independent effects of nutrients on eBMD, accounting for the potential confounding effects of these factors. The genetic variants used in the MVMR were fixed to the reported IVs of nutrients. In addition, we explored the causal effects of nutrients on sex-specific eBMD.

To further supplement the information derived from two-sample MR, we performed a one-sample MR analysis to investigate whether causal relationships also exist between diet-derived nutrient intake and eBMD using the individual-level genotyping data from the UK Biobank cohort. We first constructed a weighted polygenic risk score (PRS) to integrate the genetic effects of candidate SNPs on the nutrients for available individual-level genotyping data, using the formula: ${PRS}_{j}=\sum_{i}^{N}{\beta }_{i}*{dosage}_{ij}$, where $N$ is the number of SNPs, ${\beta }_{i}$ is the previously published effect size of variant $i$, and ${dosage}_{ij}$ is the number of copies of SNP $i$ (0, 1, 2) in the genotype of individual $j$[53]. Then, we used the ratio of coefficients method [54] to elucidate causal relationships across all participants, as well as in women and men. All analyses were adjusted for age, sex, assessment center, assessment month, relevant vitamin or mineral supplement, BMI, smoking status, alcohol consumption, genotype array, and the top 40 genetic principal components. To further investigate the non-linear relationship, we employed a doubly-ranked stratification method [55] to divide the population into five strata, calculated linear MR estimates for each stratum to obtain localized average causal effects and fitted these using a fractional polynomial model [56]. Additional adjustments for age-squared, age-by-sex interaction, and age-squared-by-sex interaction were made based on the covariates adjusted for in the linear analysis.

A suggestive significance was defined as *P* < 0.05, while Bonferroni-corrected significance was set as *P* < 0.05/(number of trait pairs) to account for multiple comparisons and ensure more reliable results. All analyses were performed using R software (v4.2.3) with main packages including “TwoSampleMR” (v0.5.5), “MRPRESSO” (v1.0), “MendelianRandomization” (v0.9.0), “cause” (v1.2.0), “MVMR” (v0.4), “OneSampleMR” (v0.1.3), “ivreg” (v0.6.2), and “SUMnlmr” (v0.0.0.9000).

## Results

### Main analysis for screening

**Fig. 2:**
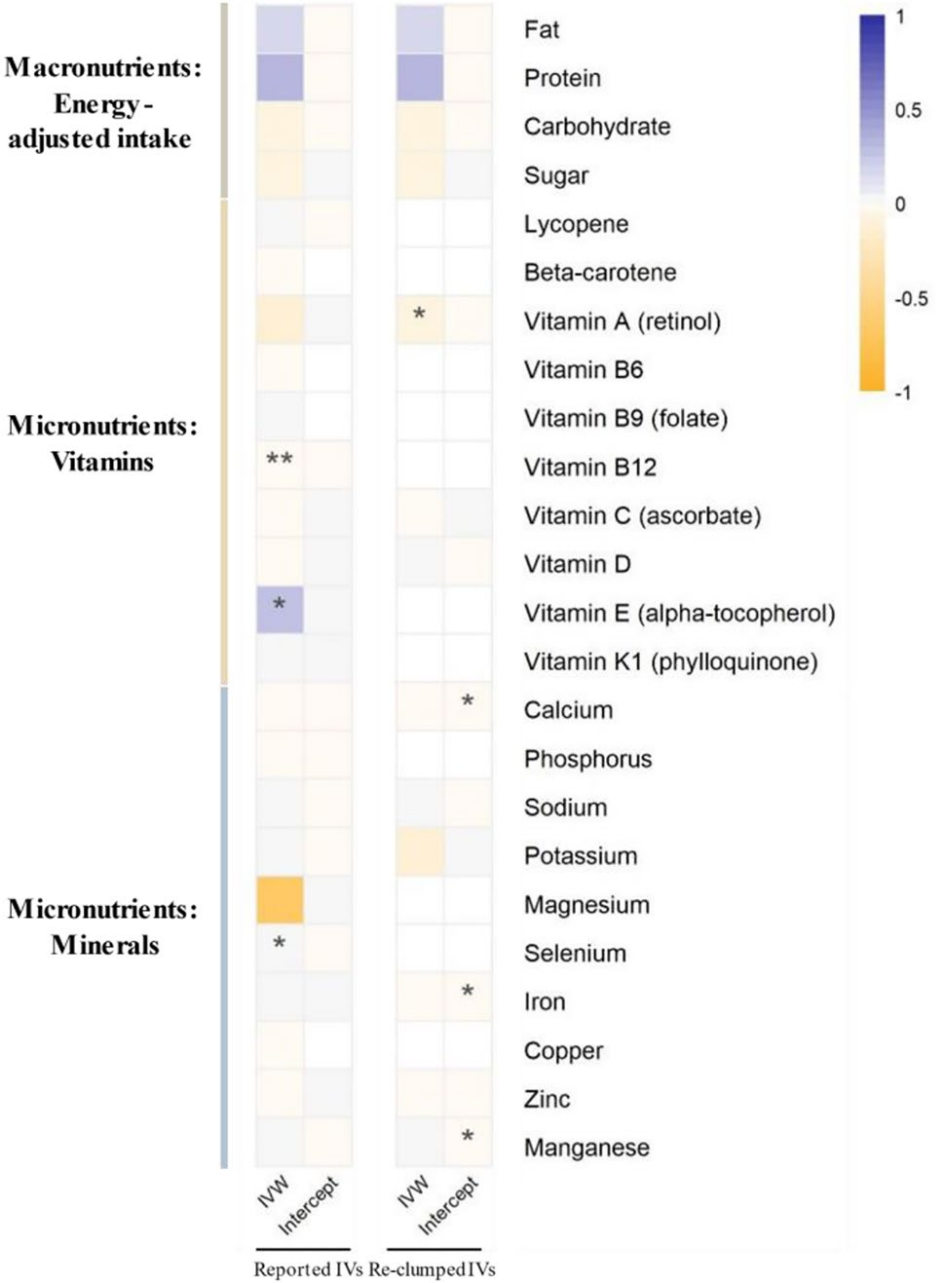
Causal relationships of nutrients and heel estimated bone mineral density (eBMD) by main MR method^#^. *MR* Mendelian randomization, *IVW* inverse variance weighted, *IVs* instrumental variables. ^#^The IVW (multiplicative random effects) and MR-Egger method. The figure was made with causal effect ($\beta$) and intercept, colored by direction (blue represents positive; orange represents negative), and shades of color represent magnitude. One asterisk (*) represents *P* < 0.05, and two asterisk (**) represent the tests that survived Bonferroni correction (*P* < 0.05/number of phenotype pairs). Completely blank cells represent no values

Significant heterogeneity was observed across almost all genetic instruments (all *P*_Cochrane’s Q_ < 0.05, except for vitamin B12, which was 0.84); therefore, we used the multiplicative random effects model of IVW (Supplementary Table 2). As shown in Fig. 2 and Supplementary Table 2, the causal effects of plasma/serum vitamin B12, vitamin E, and selenium on eBMD were suggestively significant using the IVW approach and showed no directional pleiotropy using the MR-Egger regression method. Specifically, their causal estimates were as follows: vitamin B12 ($\beta$_IVW_ = − 0.020, 95% CI = [− 0.028, − 0.011], *P*_IVW_ = 3.71 × 10^–6^, *P*_intercept_ = 0.35), vitamin E ($\beta$_IVW_ = 0.277, 95% CI = [0.024, 0.530], *P*_IVW_ = 3.22 × 10^–2^, *P*_intercept_ = 0.14), and selenium ($\beta$_IVW_ = 0.023, 95% CI = [0.007, 0.040], *P*_IVW_ = 5.37 × 10^–3^, *P*_intercept_ = 0.37). By using available re-clumped IVs, we further found a causal effect between vitamin A and eBMD ($\beta$_IVW_ = − 0.054, 95%CI = [− 0.104, − 0.003], *P*_IVW_ = 3.70 × 10^–2^, *P*_intercept_ = 0.23) passing the screening test (Fig. 2 and Supplementary Table 5). In contrast, causal effects between other nutrients and eBMD were not observed by either the IVW approach supplemented by MR-Egger regression or the Wald ratio method (all *P*-values > 0.05), using reported or re-clumped IVs.

### Sensitivity, sex-specific, and one-sample MR analyses

For the four key nutrients (i.e., vitamin A, vitamin B12, vitamin E, and selenium) with suggestive evidence of a causal effect on eBMD in the main analysis, we performed the sensitivity analysis to further verify the robustness of these results. Additionally, we performed the sex-specific analysis to investigate sex-specific causal associations and the one-sample MR analysis to explore whether similar causal relationships also exist between diet-derived nutrient intake and eBMD.

As shown in Table 2 and Supplementary Table 6** ~ **8, the causal effects of the above four nutrients on eBMD were considered robust, as all sensitivity analyses yielded directionally consistent results. Specifically, (I) the weighted median approach exhibiting stronger robustness against invalid IVs yielded similar results with IVW; (II) the MR-PRESSO approach (at least four IVs are required) provided similar results with IVW after identifying and removing outlying IVs (vitamin E could not be evaluated by MR-PRESSO due to having only three IVs); (III) the IVW approach (without pleiotropic SNPs) maintained directional consistency after removing pleiotropic IVs linked to potential confounders (vitamin E could not be evaluated as all three IVs were pleiotropic); (IV) the leave-one-out method indicated the causal effects of the four nutrients on eBMD were not influenced by a single IV. However, only the causal effects of vitamin B12 and selenium on eBMD remained significant after Bonferroni correction (*P* < 1.25 × 10^–2^, i.e., 0.05/4).

**Table 2:** Casual relationships of the four nutrients (suggestively significant, i.e., *P* < 0.05, by main MR method^#^) and heel estimated bone mineral density (eBMD) by two-sample MR method

Exposure	MR approach^##^	Heel estimated bone mineral density (eBMD)				
		Number of IVs	Effect (96% CI)	*P*-value	MR-Egger intercept (*P*-value)	*P*-value for Cochrane’s Q
Vitamin A (retinol)^△^	IVW	5	− 0.054 (− 0.104–0.003)	**3.70 × 10**^**–2**^	− 0.009 (0.23)	3.07 × 10^–3^
	MR-Egger regression	5	0.059 (− 0.093–0.211)	0.50		
	Weighted median	5	− 0.057 (− 0.096–0.018)	**3.77 × 10**^**–3**^		
	MR-PRESSO	3	− 0.053 (− 0.077–0.029)	**5.00 × 10**^**–2**^		
	IVW (without pleiotropic SNPs)	4	− 0.049 (− 0.115–0.017)	0.14		
	MVMR (adjust for smoking status)	5	− 0.130 (− 0.198–0.061)	**2.13 × 10**^**–4**^		
	MVMR (adjust for alcohol assumption)	5	− 0.054 (− 0.119–0.010)	0.10		
Vitamin B12^*^	IVW	9	− 0.020 (− 0.028–0.011)	**3.71 × 10**^**–6**^	− 0.002 (0.35)	0.84
	MR-Egger regression	9	− 0.009 (− 0.034–0.016)	0.52		
	Weighted median	9	− 0.020 (− 0.034–0.006)	**5.04 × 10**^**–3**^		
	MRPRESSO	9	− 0.020 (− 0.028–0.011)	**3.71 × 10**^**–6**^		
	IVW (without pleiotropic SNPs)	6	− 0.013 (− 0.028–0.002)	0.10		
	MVMR (adjust for BMI)	9	− 0.023 (− 0.038–0.009)	**1.71 × 10**^**–3**^		
	MVMR (adjust for smoking status)	9	− 0.020 (− 0.032–0.008)	**1.38 × 10**^**–3**^		
	MVMR (adjust for alcohol assumption)	9	− 0.018 (− 0.031–0.004)	**9.70 × 10**^**–3**^		
Vitamin E (alpha-tocopherol)^▽△^	IVW	3	0.277 (0.024–0.530)	**3.22 × 10**^**–2**^	0.045 (0.14)	8.31 × 10^–5^
	MR-Egger regression	3	− 1.079 (− 1.698–0.460)	0.18		
	Weighted median	3	0.161 (− 0.025–0.346)	**3.95 × 10**^**–2**^		
	MVMR (adjust for smoking status)	3	0.598 (0.426–0.770)	**1.00 × 10**^**–11**^		
	MVMR (adjust for alcohol assumption)	3	0.281 (− 0.365–0.927)	0.39		
Selenium^△*^	IVW	12	0.023 (0.007–0.040)	**5.37 × 10**^**–3**^	− 0.004 (0.37)	1.04 × 10^–4^
	MR-Egger regression	12	0.048 (− 0.006–0.103)	0.11		
	Weighted median	12	0.026 (0.012–0.040)	**1.67 × 10**^**–4**^		
	MRPRESSO	10	0.023 (0.009–0.038)	**1.00 × 10**^**–2**^		
	IVW (without pleiotropic SNPs)	9	0.029 (0.016–0.042)	**1.97 × 10**^**–5**^		
	MVMR (adjust for BMI)	12	0.022 (0.004–0.040)	**1.79 × 10**^**–2**^		
	MVMR (adjust for alcohol assumption)	12	0.017 (0.001–0.033)	**4.23 × 10**^**–2**^		

*MR* Mendelian randomization, *IVs* instrumental variables, *CI* confidence interval, *IVW* inverse variance weighted, *MRPRESSO* MR-Pleiotropy Residual Sum and Outlier, *SNPs* single nucleotide polymorphisms, *MVMR* multivariable MR, *BMI* body mass index, *GWAS* genome wide association study

^#^The inverse variance weighted (multiplicative random effects) and MR-Egger method

^##^The MRPRESSO method is used when it is greater than or equal to 4

^▽^All three IVs of vitamin E were pleiotropic, so the IVW (without pleiotropic SNPs) was not conducted

^△^The original GWAS summary statistics for vitamin A and vitamin E were already adjusted for BMI, and the GWAS data for selenium were already adjusted for smoking status, eliminating the necessity for further adjustment in MVMR for these factors

A suggestive significance was defined as *P* < 0.05; a statistical significance^*^ was defined as *P* < 1.25 × 10^–2^ (0.05/4, number of trait pairs) to account for multiple comparisons through Bonferroni

After adjusting for BMI, smoking status, and alcohol consumption using MVMR, we observed robust and independent effects of two genetically predicted nutrients on eBMD, specifically, vitamin B12 ($\beta$ = − 0.023 ~  − 0.018, *P* = 1.38 × 10^–3^ ~ 9.70 × 10^–3^) and selenium ($\beta$ = 0.017 ~ 0.022, *P* = 1.79 × 10^–2^ ~ 4.23 × 10^–2^) (Table 2). In contrast, the causal effects of vitamin A and vitamin E on eBMD were independent of smoking status but not alcohol consumption (Table 2). Notably, the original GWAS summary statistics for selenium were already adjusted for smoking status, and those for vitamin A and vitamin E were already adjusted for BMI, eliminating the necessity for further adjustment in MVMR for these factors.

**Fig. 3:**
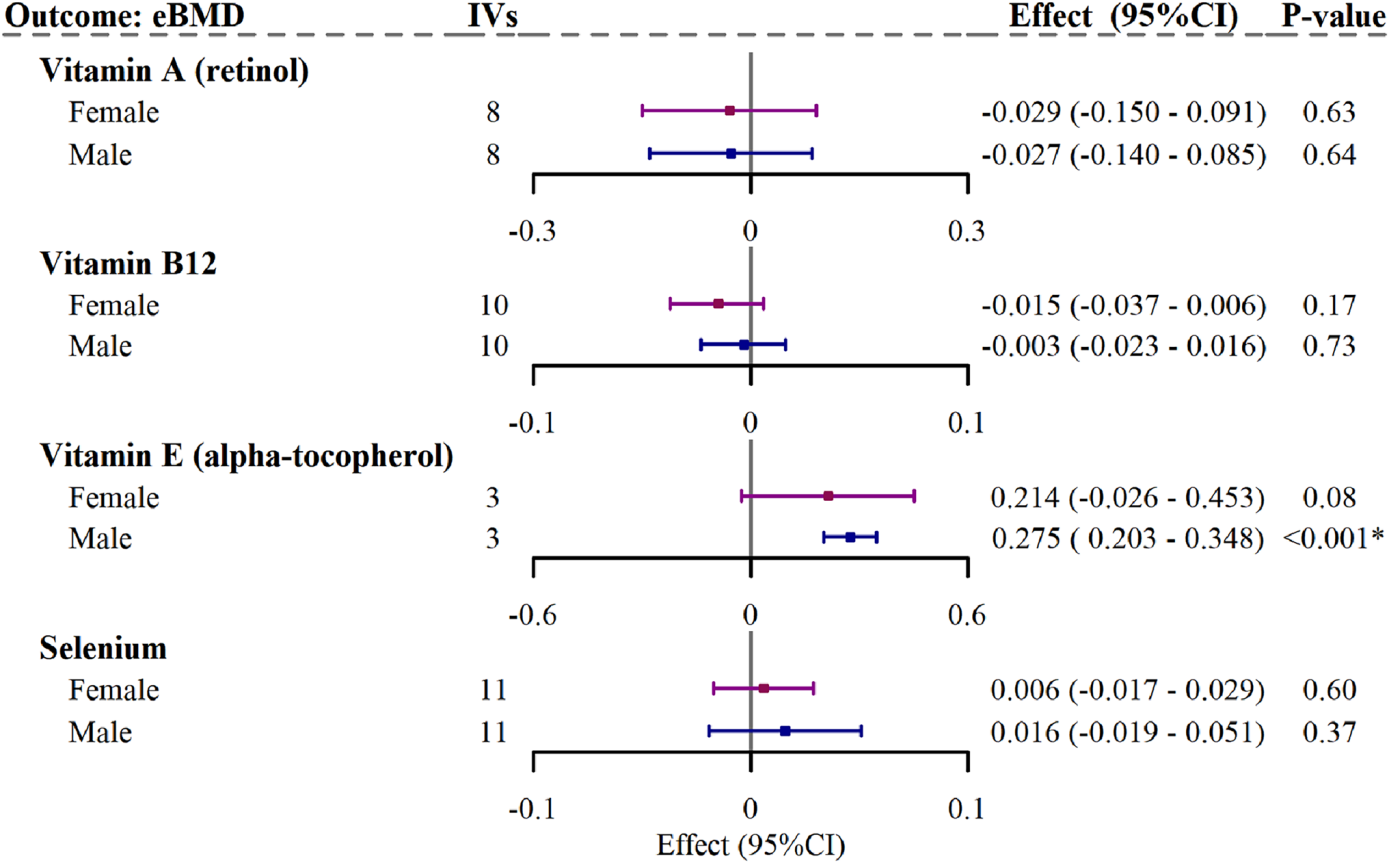
Causal relationships of the four nutrients (suggestively significant, i.e., *P* < 0.05, by main MR method^#^) and sex-specific heel bone mineral density (eBMD) by the IVW method. *IVs* instrumental variables, *CI* confidence interval, *IVW* inverse variance weighted. ^#^The inverse variance weighted (multiplicative random effects) and MR-Egger method. Different colors represent different genders, i.e. purple for female and blue for male. A suggestive significance was defined as *P* < 0.05; a statistical significance^*^ was defined as *P* < 6.25 × 10^–3^ (0.05/8, number of trait pairs) to account for multiple comparisons by the Bonferroni correction

As shown in Fig. 3 and Supplementary Table 9, only the causal relationship between vitamin E and eBMD exhibited differences in sex-specific analysis. The causal effects between vitamin E and eBMD were observed only in the males ($\beta$= 0.275, 95%CI = [0.203, 0.348], *P* = 9.81 × 10^–14^), but not in the females ($\beta$= 0.214, 95%CI = [− 0.026, 0.453], *P* = 0.08).

The basic characteristics of 52,851 participants from the UK Biobank cohort were listed in Supplementary Table 10. As shown in Table 3, unlike the results of circulating levels of nutrients, genetically predicted diet-derived nutrients did not exhibit causal associations with eBMD in the overall population or either sex ($\beta$ = − 2.894 ~ 0.444, all *P*-values = 0.10 ~ 0.97 > 0.05). Furthermore, as shown in Table 3 and Supplementary Table 11, no non-linear relationships were observed between the four genetically predicted diet-derived nutrients and eBMD (all *P*-values for heterogeneity = 0.34 ~ 0.96 > 0.05, and nonlinearity = 0.07 ~ 0.61 > 0.05).

**Table 3:** Causal relationships of the four dietary-derived nutrients (suggestively significant, i.e., *P* < 0.05, by main MR method^#^) and heel estimated bone mineral density (eBMD) by one-sample MR method^##^ (*N* = 52,851; 55% female)

Exposure	Group	Effect (96% CI)	*P*-value	*P*-value for heterogeneity	*P*-value for nonlinearity
Vitamin A	Female	0.174 (− 0.042–0.391)	0.11	0.37	0.26
	Male	0.058 (− 0.157–0.272)	0.60		
	All	0.119 (− 0.023–0.261)	0.10		
Vitamin B12	Female	− 0.021 (− 0.473–0.430)	0.93	0.34	0.07
	Male	− 0.030 (− 0.470–0.411)	0.89		
	All	0.006 (− 0.315–0.327)	0.97		
Vitamin E	Female	0.444 (− 0.386–1.273)	0.29	0.71	0.36
	Male	0.153 (− 0.299–0.605)	0.51		
	All	− 2.894 (− 66.65–60.86)	0.93		
Selenium	Female	− 0.334 (− 1.132–0.463)	0.41	0.96	0.61
	Male	− 0.016 (− 1.010–0.977)	0.97		
	All	− 0.239 (− 0.849–0.371)	0.44		

*MR* Mendelian randomization, *N* sample size, *CI* confidence interval, *BMI* body mass index

^#^The inverse variance weighted (multiplicative random effects) and MR-Egger method

^##^The linear and nonlinear MR were conducted using the ratio of coefficients method and the fractional polynomial model, respectively. Both methods were adjusted for age, sex (only the total participants), assessment center, assessment month, relevant vitamin or mineral supplement, BMI, smoking status, alcohol consumption, genotype array, and the top 40 genetic principal components. Additional adjustments for age-squared, age-by-sex interaction, and age-squared-by-sex interaction were made in the non-linear analysis

## Discussion

This study utilized the largest available GWAS summary statistics to date and a comprehensive MR analytical framework to systematically investigate the causal effects of energy-adjusted macronutrient intake (fat, protein, carbohydrate, and sugar) and circulating levels of 20 micronutrients (ten each for vitamins and minerals) on eBMD. Our findings suggest that appropriate levels of plasma vitamin B12 and adequate levels of serum selenium may enhance eBMD and reduce osteoporosis risk, highlighting the importance of maintaining optimal levels of these nutrients to delay bone loss and promote bone health.

To the best of our knowledge, this is the first study to investigate the causal effects of energy-adjusted macronutrient intake (**fat, protein, carbohydrate, and sugar**) on eBMD. Similarly, the causal associations between 10 out of 20 circulating micronutrients (i.e., **lycopene, beta-carotene, vitamin C, vitamin D, vitamin K1, sodium, potassium, copper, zinc, and manganese**) and eBMD were also investigated for the first time. In general, no significant causal effects were found. Using osteoporosis risk as the outcome (self-reported or diagnosed), previous MR studies have also suggested no causal effects of plasma/serum lycopene, beta-carotene, vitamin C, vitamin D, copper, and zinc on osteoporosis risk [10, 11, 57], which is to some extent consistent with our results. In addition, for six of the other 10 micronutrients, by using larger GWAS summary statistics and more IVs, we re-confirmed the lack of causal effects for **vitamin B6, vitamin B9, calcium, phosphorus, magnesium, and iron** on eBMD, aligning with previous MR studies [9, 12, 13]. These negative findings may be due to inadequate statistical power and require further validation.

Previous observational studies have reported conflicting associations, with opposite directions, between serum **vitamin A (retinol)** and BMD [58, 59]. Using two IVs derived from a small-scale GWAS summary statistic involving 8,902 participants [60], an MR study found a positive causal association between serum retinol and eBMD ($\beta$ = 0.130, *P* = 1.00 × 10^–2^) with male specificity ($\beta$ = 0.310, *P* = 1.00 × 10^–2^) [12]. Nevertheless, the limited number of IVs may compromise the precision of causal estimates due to insufficient power. By utilizing eight IVs from a larger GWAS summary statistic involving 17,268 participants, we found a negative causal effect of plasma retinol on eBMD with no gender specificity. In contrast, we found a positive causal effect between serum **vitamin E (alpha-tocopherol)** and eBMD with male specificity using three IVs, consistent with previous MR studies [8, 12], suggesting the reproducibility of results. However, after Bonferroni correction, the causal effects of these two vitamins on eBMD were no longer significant. While independent of smoking status, the causal effects were influenced by alcohol consumption, suggesting potential bias. Furthermore, the causal relationships were also not observed using individual-level data on diet-derived vitamin A and vitamin E intake. Therefore, the causal relationships between these two vitamins (i.e., vitamin A and vitamin E) and eBMD should be viewed with caution, requiring further validation with larger available genetic and epidemiological data.

A previous meta-analysis of cross-sectional studies involving 674 to 1,037 female participants showed no association between serum/plasma **vitamin B12** and BMD at the femoral neck, lumbar spine, and total hip [61]. However, a subsequent meta-analysis of six case–control studies revealed a statistically significant difference in levels of vitamin B12 between the postmenopausal osteoporosis group (n = 288) and the control group (n = 361) [62]. These inconsistent results may stem from inherent limitations in observational studies, such as the potential impact of confounders and reverse causality. Using the MR method, a recent study found no causal relationship between plasma vitamin B12 and eBMD [13]. By utilizing reported IVs based on a stricter criterion of vitamin B12 GWAS and a largely augmented eBMD GWAS, we found a robust negative causal effect of plasma vitamin B12 on eBMD with no sex specificity. Moreover, this causal effect remained significant after Bonferroni correction and was independent of BMI, smoking status, and alcohol consumption, suggesting that unobserved pleiotropic effects and confounders did not bias our results. Although no causal relationship between diet-derived vitamin B12 intake and eBMD was found using individual-level data in the UK Biobank cohort, a consistent direction of causal effects was observed. The detrimental effects of excess vitamin B12 on bone health remain unclear but may be related to hypermethylation due to the overpromotion of one-carbon metabolism [63]. Previous research has indicated that DNA methylation can regulate the differentiation and function of osteoblasts/osteoclasts by controlling the expression of related genes, thereby influencing the balance between bone formation and resorption, and mediating the occurrence and development of osteoporosis [64, 65]. Overall, this finding highlights the significance of maintaining plasma levels of vitamin B12 within an appropriate range for bone health.

In addition, we found that higher levels of serum **selenium** were robustly associated with an increased eBMD, which remained significant after Bonferroni correction and was independent of BMI and alcohol consumption. The results are consistent with a recently published MR [12] that used a relatively small-scale GWAS summary statistic for eBMD. Similarly, a recent meta-analysis of 19 observational studies involving 69,672 participants also found significantly positive associations between dietary selenium intake ($\beta$ = 0.040, *P* = 3.00 × 10^–2^) as well as serum selenium ($\beta$ = 0.130, *P* = 5.00 × 10^–2^) and BMD [66]. However, we found no causal relationship between diet-derived selenium intake and eBMD using individual-level data in the UK Biobank cohort, which might be due to insufficient power caused by the small sample size and required further investigation and validation. Selenium, known for its antioxidant properties, has been implicated to potentially protect against osteoarthritis [67] and rheumatoid arthritis [68], two prevalent types of arthritis that can cause pain and joint damage. Given that oxidative stress has also been suggested as destructive to osteoporosis [69], it is plausible to speculate that selenium has a protective effect on eBMD and decreases osteoporosis risk through its antioxidant action, highlighting the importance of maintaining adequate levels of serum selenium for bone health.

Taken together, our research highlights the importance of maintaining appropriate levels of plasma vitamin B12 and adequate levels of serum selenium as an effective lifestyle intervention to enhance eBMD and reduce osteoporosis risk. Our results exhibit significant implications for clinical practice and public health policy. Specifically, our findings indicate that high levels of plasma vitamin B12 in populations at high risk of osteoporosis should perhaps be avoided, while maintaining adequate levels of serum selenium may be beneficial. Therefore, we advocate for maintaining optimal circulating levels of these two nutrients to delay bone loss and promote bone health. Future research is warranted to confirm these effects across different populations and to establish clinical recommendations and potential causal mechanisms.

We acknowledge several potential limitations. First, all analyses were performed using European participants only, limiting the generalizability of these results to other populations. Second, despite using the hitherto largest available GWAS summary statistics to extract genetic variants, the IVs explained only a small amount of phenotypic variance, resulting in limited statistical power to detect weak to moderate associations for many macro and micronutrients. Third, using energy-adjusted macronutrient intake and diet-derived nutrient intake might be influenced by measurement errors due to recall bias in self-report questionnaires [70]. Fourth, there was sample overlap between certain micronutrients (i.e., vitamin D, calcium, sodium, potassium) and eBMD, as all data were from the UKB cohort. By comparing the results of the IV-based MR approach (IVW) and the genome-wide variant-based MR approach (CAUSE [71]), we assumed that the causal associations were very less likely to be biased by sample overlap (*P*_IVW_ = 0.14 ~ 0.93 and *P*_CAUSE_ = 0.06 ~ 0.36, all *P*-values > 0.05; Supplementary Table 11). Fifth, the GWAS summary statistics for serum vitamin A and vitamin E were adjusted for BMI, and those for selenium were adjusted for smoking status, which could potentially introduce collider bias in MR estimates [72]. Thus, GWAS summary statistics for these two exposures without BMI adjustment are needed. Finally, a potential dose–response effect of nutrients on eBMD could not be ruled out, necessitating more in-depth research to elucidate this relationship. Therefore, these analyses should be repeated as larger GWAS summary statistics and GWAS summary statistics of other populations become available.

## Conclusions

In conclusion, our findings underscore the importance of maintaining optimal levels of plasma vitamin B12 and serum selenium for promoting bone health and delaying bone loss. These results highlight the potential of targeted nutritional interventions to improve eBMD and reduce osteoporosis risk. Future research should prioritize validating these findings in large-scale cohorts and diverse populations, elucidating the underlying mechanisms, and evaluating the efficacy of dietary interventions. Such efforts are essential for developing evidence-based strategies to enhance bone health and alleviate the global burden of osteoporosis.

## Supplementary Information

Below is the link to the electronic supplementary material.

Supplementary file1 (XLSX 180 KB)

Supplementary file2 (DOCX 33 KB)

## Data Availability

This study did not generate new datasets or codes. Both publicly available summary statistics and individual participant cohort data were used. Full information on how to access UK Biobank data can be found on its website (https://www.ukbiobank.ac.uk/). More details of the approaches as well as the codes are available at https://mrcieu.github.io/TwoSampleMR/ (TwoSampleMR), https://www.cog-genomics.org/plink/1.9/ (PLINK), https://jean997.github.io/cause/ (cause), https://github.com/rondolab/MR-PRESSO (MRPRESSO), https://wspiller.github.io/MVMR/ (MVMR), https://github.com/remlapmot/OneSampleMR (OneSampleMR), and https://github.com/amymariemason/SUMnlmr (SUMnlmr).
